# The trisomy 18 syndrome

**DOI:** 10.1186/1750-1172-7-81

**Published:** 2012-10-23

**Authors:** Anna Cereda, John C Carey

**Affiliations:** 1 Ambulatorio Genetica Clinica Pediatrica, Clinica Pediatrica Universita Milano Bicocca, Fondazione MBBM A.O, S, Gerardo Monza, Italy; 2 Division of Medical Genetics, Department of Pediatrics, University of Utah, 419 Wakara Way Suite 213, Salt Lake City, UT, 84108, USA

## Abstract

The trisomy 18 syndrome, also known as Edwards syndrome, is a common chromosomal disorder due to the presence of an extra chromosome 18, either full, mosaic trisomy, or partial trisomy 18q. The condition is the second most common autosomal trisomy syndrome after trisomy 21. The live born prevalence is estimated as 1/6,000-1/8,000, but the overall prevalence is higher (1/2500-1/2600) due to the high frequency of fetal loss and pregnancy termination after prenatal diagnosis. The prevalence of trisomy 18 rises with the increasing maternal age. The recurrence risk for a family with a child with full trisomy 18 is about 1%.

Currently most cases of trisomy 18 are prenatally diagnosed, based on screening by maternal age, maternal serum marker screening, or detection of sonographic abnormalities (e.g., increased nuchal translucency thickness, growth retardation, choroid plexus cyst, overlapping of fingers, and congenital heart defects ). The recognizable syndrome pattern consists of major and minor anomalies, prenatal and postnatal growth deficiency, an increased risk of neonatal and infant mortality, and marked psychomotor and cognitive disability. Typical minor anomalies include characteristic craniofacial features, clenched fist with overriding fingers, small fingernails, underdeveloped thumbs, and short sternum. The presence of major malformations is common, and the most frequent are heart and kidney anomalies. Feeding problems occur consistently and may require enteral nutrition.

Despite the well known infant mortality, approximately 50% of babies with trisomy 18 live longer than 1 week and about 5-10% of children beyond the first year. The major causes of death include central apnea, cardiac failure due to cardiac malformations, respiratory insufficiency due to hypoventilation, aspiration, or upper airway obstruction and, likely, the combination of these and other factors (including decisions regarding aggressive care). Upper airway obstruction is likely more common than previously realized and should be investigated when full care is opted by the family and medical team.

The complexity and the severity of the clinical presentation at birth and the high neonatal and infant mortality make the perinatal and neonatal management of babies with trisomy 18 particularly challenging, controversial, and unique among multiple congenital anomaly syndromes. Health supervision should be diligent, especially in the first 12 months of life, and can require multiple pediatric and specialist evaluations.

## Disease names and synonyms

Trisomy 18, Edwards syndrome

## Definition

**Figure 1:**
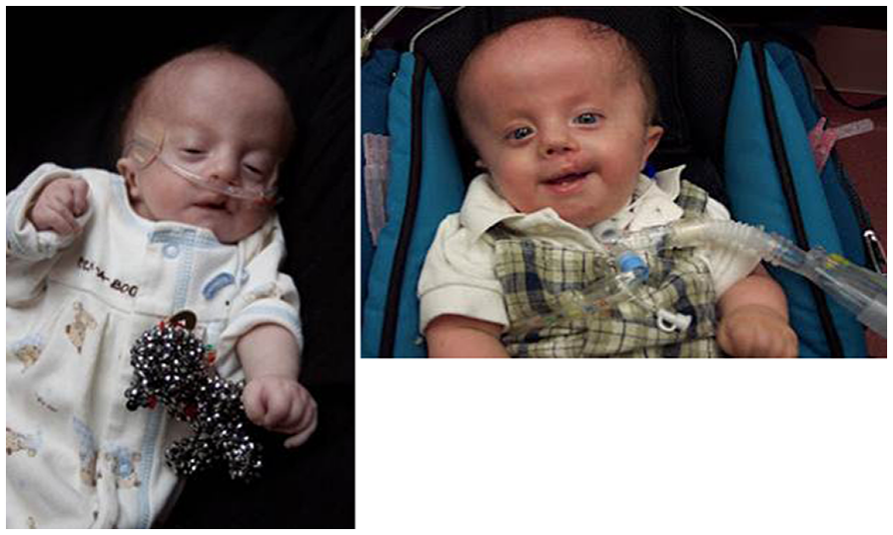
**A boy with full trisomy 18 in early infancy and at one year.** Note the characteristic hand feature with the over-riding fingers, the tracheostomy, and his engaging smile. He is now over 2 years of age and is quite stable medically, gaining weight, sitting up, and participating in the many activities of his family.

The trisomy 18 syndrome, also known as Edwards syndrome, is a common autosomal chromosomal disorder due to the presence of an extra chromosome 18. The first reported infants were described in 1960 by Edwards et al. and Smith et al. [1,2]. The syndrome pattern comprises a recognizable pattern of major and minor anomalies, an increased risk of neonatal and infant mortality, and significant psychomotor and cognitive disability. The main clinical features represent the clues for the diagnosis in the perinatal period and include prenatal growth deficiency, characteristic craniofacial features, distinctive hand posture (overriding fingers, see Figure 1), nail hypoplasia, short hallux, short sternum, and major malformations (particularly involving the heart). The demonstration of an extra chromosome 18, or less commonly a partial trisomy of the long arm of chromosome 18, on the standard G-banded karyotype allows for confirmation of the clinical diagnosis. A small portion of patients (less than 5% in population studies cited below) have mosaicism of trisomy 18; they show an extremely variable phenotype.

## Epidemiology

Trisomy 18 is the second most common autosomal trisomy syndrome after trisomy 21.

Several population studies have been performed in different countries including Australia, Europe and North America that estimate the prevalence of trisomy 18 [3-9]. On the basis of these investigations the live birth prevalence of trisomy 18 ranges from 1/3600 to 1/10,000 with the best overall estimate in liveborns as 1 in 6,000 [3,6].

It is well known that trisomy 18 pregnancies have a high risk of fetal loss and stillbirth [10,11]; furthermore, currently most diagnoses are made in the prenatal period based on screening by maternal age or maternal serum marker screening and amniocentesis, followed by pregnancy termination in a significant percentage of cases [9]. Because of this, the overall prevalence (considering stillborn infants, terminated pregnancies, and liveborn infants) of trisomy 18 would be expected to be higher than live birth prevalence. A seminal population study in the United Kingdom in 1996 reported an overall prevalence of 1/4272 and a liveborn prevalence of 1/8333 [4]; the overall frequency detected in Hawaii from a similar study was 1/2123 with a liveborn frequency of 1/7900 [5]. Recent investigations showed an increase of the overall prevalence of trisomy 18 over the last 20 years due to increased maternal age [9]; however, a decrease of liveborn frequency was observed because of the increased use of prenatal diagnosis and the high rate of pregnancy termination after the prenatal diagnosis [7,9]. In these more recent studies overall prevalence was estimated as 1/2500 in United States [7] and as 1/2600 in United Kingdom [9]; liveborn prevalence was estimated as 1/8600 in United States [7] and as 1/10,000 in United Kingdom [9].

The prevalence at birth is higher in females compared to males (F:M %, 60.4), but this discordance is not present if the sex ratio is calculated among fetuses electively terminated (F:M % 48:51.) [7]. Moreover the frequency of fetal loss is higher for males compared to females [10,11]. Furthermore, liveborn females showed better survival compared to males [4,6].

## Etiology and pathogenesis

The trisomy 18 (or Edwards syndrome) phenotype results from full, mosaic, or partial trisomy 18q [4,12-15]. Complete or full trisomy 18 is the most common form (about 94% of cases); in this situation every cell contains three entire copies of chromosome 18.

Most authorities have suggested that the extra chromosome is present because of nondisjunction. In parent-of-origin analyses the extra chromosome is most often of maternal origin, the result of an error during the segregation of chromosomes in meiosis or postzygotic mitosis. About 50% of the nondisjunctional errors in oogenesis occur in meiosis II, unlike other human trisomies where the malsegregation is more frequent in meiosis I [16-19]. In the minority of cases in which the extra chromosome has a paternal origin, the error is the result of a postzygotic error. The cause of nondisjunction is unknown. Recently a higher prevalence of methylene tetrahydrofolate reductase gene (*MTHFR*) polymorphisms in mothers of trisomy 18 fetuses compared with other groups was reported [20] but this result has not been replicated.

As in the other common autosomal trisomies, the frequency of nondisjunctional errors increases with advancing maternal age. Savva et al., studied the maternal age specific live birth prevalence of trisomy 18: the frequency is constant until age 30, then increases exponentially before beginning to become constant again at age 45 [21]. The observed increased overall prevalence of trisomy 18 in the last years is likely due to changes in the maternal age distribution during this time period [9,21]. A small positive association of paternal age with trisomy 18, similar to that observed in Down syndrome, has also been observed [22].

In individuals carrying mosaic trisomy 18 (less than 5% of cases), both a complete trisomy 18 and a normal cell line exist. The phenotype is extremely variable, ranging from complete trisomy 18 phenotype with early mortality to apparently phenotypically normal adults, in which the mosaicism is detected after the diagnosis of complete trisomy 18 in a child [23-27]. There is no correlation between the percentage of trisomy 18 cells in either blood cells or skin fibroblasts and the severity of clinical manifestations and intellectual disabilities [24]. Tucker et al., [24] provided a comprehensive review of all published cases of trisomy 18 mosaicism in their recent paper and reported on 3 new cases.

In the partial trisomy form only a segment of the chromosome 18 long arm is present in triplicate, often resulting from a balanced translocation or inversion carried by one parent. This type of trisomy accounts for approximately 2% of cases presenting with the Edwards phenotype. The location and the extent of the triplicated segment and the possible associated deletion of genomic material due to unbalanced translocation can explain the variable phenotype associated with partial trisomy [12].

The region of long arm of chromosome 18 extending from q11.2 has been proposed as the critical region for trisomy 18 phenotype, but some controversial data have been reported [28,29]. Boghosian-Sell et al. hypothesized the presence of two critical regions along the long arm of chromosome 18: one proximal region lying within 18q12.1-18q21.2 and another one more distal lying within 18q22.33-18qter [29]. The same authors reported two patients with trisomy of 18q11.2 to terminus not showing the complete pattern of trisomy 18; the patients had better survival and growth. Therefore, some role for genes on the short arm or 18q11.1 region in the expression of full phenotype cannot be excluded.

## Antenatal diagnosis

Currently in the North America and Europe most cases of trisomy 18 are prenatally diagnosed, based on screening by maternal age, maternal serum marker screening, or detection of sonographic abnormalities during the second and third trimester [9,30]. The prenatal diagnosis of trisomy 18 leads to the decision of pregnancy termination in 86% of cases [9]. Knowledge of the survival where termination is not chosen is important as well, because the parents will seek this information and this knowledge can influence the management at the time of delivery and in the neonatal period [12].

First trimester non invasive screening based on maternal age, serum markers and sonographic “soft markers” demonstrated high sensitivity for diagnosis of trisomy 18 [31-33], and it is now being applied routinely. The levels of human chorionic gonadotropin, unconjugated estriol, and alpha-fetoprotein are significantly lower in pregnancies with trisomy 18 compared to normal pregnancy [31]. The most common soft sonographic markers detected in the late first/early second trimester are the increased nuchal translucency thickness and the absence or hypoplasia of the nasal bone [34-36]; the screening by assessment of nuchal fold and nasal bone identifies 66.7% of cases with trisomy 18 (and 13) [36]. By including the evaluation of reversed flow in the ductus venosus and the tricuspid valve regurgitation, the detection rate increases to 83.3% [36]. Furthermore, some structural anomalies can be detected by ultrasound screening during the first trimester; the most common are omphalocele (21%), abnormal posturing of the hands (6%), megacystis (4%) and abnormal four-chamber view of the heart (4%) [35]. Early-onset fetal growth retardation can be detected in 26% of cases [36], but becomes more evident in the second trimester [30,37]. The detection rate of combined late first trimester screening (nuchal translucency, pregnancy-associated plasma protein and free beta-hCG) and second trimester quadruple screening (serum alpha-fetoprotein, total hCG, unconjugated estriol and inhibin A) is at least 78% sensitive [32,33].

Many studies have been published in the last 15 years regarding the prenatal pattern of ultrasound findings in trisomy 18 fetuses in the second and third trimester [30,35-39]. One or more sonographic anomalies are detected in over 90% of fetuses; two or more abnormalities are present in 55% of cases [38]. The prenatal sonographic pattern of trisomy 18 is characterized by growth retardation, polyhydramnios, “strawberry-shaped” cranium (brachycephaly and narrow frontal cranium), choroid plexus cyst, overlapping of hands fingers (second and fifth on third and fourth respectively), congenital heart defects, omphalocele, and single umbilical artery [30,35-39]. The prevalence of growth retardation and polyhydramnios increases with gestational age: 28% and 29% in the second trimester and 87% and 62% in the third trimester, respectively [37]. More than 30% of fetuses show hands abnormalities [39], and one third of cases have a single umbilical artery [37]. Furthermore, the mothers often noted a decrease in fetal movement compared to their normal pregnancies [37]. Choroid plexus cyst (CPC) is detected in about 50% of trisomy 18 fetuses [39]; in the most of cases (80-90%) it is associated with other sonographic anomalies [37,39], but in a small percentage of pregnancies (11% according to Cho et al. 2010) carrying trisomy 18 fetus, CPC can be the only abnormality detected at ultrasound screening. Choroid plexus cyst can be also a transitory finding in normal fetuses; it has been reported that, among fetuses that show CPC at second trimester sonographic screening, only about 5% have trisomy 18 [37,40,41]. Because of these reasons, there is not a clear consensus in the medical literature on whether to offer amniocentesis after the discovery of choroid cyst, particularly when it is an isolated finding [37,42-46].

Trisomy 18 pregnancies have a high risk of fetal loss and stillbirth [10,11,37]. The probability of survival to term increases with the increase of gestational age: 28% at 12 weeks, 35% at 18 weeks and 41% at 20 weeks [10]. Fetal losses are uniformly distributed throughout gestation after 24 weeks without a clustering of fetal demises at a particular gestational age [11,37]. Cases detected by abnormal sonographic findings are more likely to result in a miscarriage or stillbirth [37]. Furthermore, the frequency of miscarriage or stillbirth is higher (up to twofold according to Niedrist et al., [47]) for males compared to females [10,47].

## Genetic counseling

When prenatal or neonatal diagnosis of trisomy 18 is made, the counseling of the family should be realistic, but not desolate. The parents can find it difficult to accept the lack of certainty of the newborn situation, but they have to be prepared for both the probability of death and the possibility of living [48]. Because the parents have to make practical decisions concerning resuscitation, surgery and life support, all options for newborn management should be explained. The complex issues regarding perinatal management are covered in more detail below.

Facilitating the family getting in touch with family support groups can be helpful: they can share experiences, thoughts and concerns regarding health problems of their children, and daily situations that they are coping with. Table 1 shows the known support groups in North America, Europe, Japan, and Australia.

**Table 1:** International parent support groups for trisomy 18

**Country**	**Support Group**	**Web site**
Australia	SOFT of Australia	http://members.optushome.com.au/softaus
Europe	Chromosome 18 Registry and Research Society (Europe)	http://www.chromosome18eur.org
France	Valentin APAC Association de Porteurs d'Anomalies Chromosomiques	http://www.valentin-apac.org
Germany	LEONA e.V. - Verein für Eltern chromosomal geschädigter Kinder	http://www.leona-ev.de
Ireland	SOFT of Ireland	http://softireland.com
Italy	SOFT Italia	http://www.trisomia.org/index.html
Japan	The Trisomy 18 Support Group	http://18trisomy.com
United Kingdom	SOFT of United Kingdom	http://www.soft.org.uk
United States	USA Support Group SOFT	http://www.trisomy.org
United States	Trisomy 18 Foundation	http://www.trisomy18.org
United States	The Chromosome 18 Registry and Research Society	http://www.chromosome18.org

The recurrence risk, for a family with a child with complete trisomy 18 is usually stated as 1% [12]. Parental mosaicism has been reported in a few cases [24-27]. Furthermore, recurrence of different trisomies in the same family has been reported [49]. Empirically calculated risks suggest that the recurrence risk seems to be less than 1%, but higher than the age-specific background risk [50,51]. The recurrence risk in families with partial trisomy 18 could be higher compared with full trisomy 18, depending on the presence of a genomic rearrangement (translocation or inversion) in one of the parents.

## Clinical description

The clinical pattern of trisomy 18 is characterized by prenatal growth deficiency, specific craniofacial features and other minor anomalies, major malformations, and marked psychomotor and cognitive developmental delay.

The growth delay starts in prenatal period and continues after the birth, and most of the time is associated with feeding problems that may require enteral nutrition. Specific growth charts for trisomy 18 are available [49] and are published on the SOFT US and UK web pages (see Table 1) for printing and placement in the child’s chart. Postnatal onset microcephaly is usually present.

Typical craniofacial features include dolichocephaly, short palpebral fissures, micrognathia, (see Figure 1) external anomalies of the ears, and redundant skin at the back of the neck.

Other characteristic clinical findings are the clenched fist with overriding fingers (index finger overlapping the third and 5th finger overlapping the 4^th^ –see Figure 1), which is particularly distinctive, small fingernails, underdeveloped thumbs, short sternum, and club feet. Presence of major malformations is common, and any organ and system can be affected. Structural heart defects occur in over 90% of infants. Table 2 summarizes the most common major (medically significant) malformations detected in trisomy18 from various sources.

**Table 2:** Common major structural malformations in the trisomy 18 syndrome

**Frequency**	**Organ**/**System**	**Prevalent type of malformation**
Common (>75%)	heart	septal defects, patent ductus arteriosus, and polyvalvular disease
Frequent (25-75%)	genitourinary	horseshoe kidney
Less frequent (5-25%)	gastrointestinal	omphalocele, esophageal atresia with tracheo-esophageal fistula, pyloric stenosis, Meckel diverticulum
	central nervous system	cerebellar hypoplasia, agenesis of corpus callosum, polymicrogyria, spina bifida
	craniofacial	orofacial clefts
	eye	microphthalmia, coloboma, cataract, corneal opacities
	limb	radial aplasia/hypoplasia

From Jones [52], Baty et al. [49].

## Differential diagnosis

The clinical pattern of trisomy 18 is quite well-defined, and it is rarely misdiagnosed [12]. There are some overlapping features with Pena-Shokeir syndrome type 1 or syndromes with fetal akinesia sequence (because of polyhydramnios and joint contractures including overriding fingers), with distal arthrogryposis type 1 (because of the similar finger positioning) and with CHARGE syndrome (because of the overlapping of major malformations). The not well characterized and co-called condition known as pseudotrisomy 18 syndrome [53] probably belongs to the group of disorders with fetal akinesia sequence.

## Natural history/prognosis

### Survival after birth and neonatal management

Perinatal and neonatal management of fetuses and newborn diagnosed with trisomy 18 is multifaceted issue for a variety of reasons: the complexity and, most of the time, the severity of the clinical presentation at birth; the need of parents and care providers to urgently make decisions in care of the baby; the inevitable ethical implications due to the well known high neonatal and infant mortality, and the significant developmental disability in the surviving children that characterize this unique (together with trisomy 13) condition.

There is a high percentage of fetuses dying during labor (38.5%), and the preterm frequency (35%) is higher compared to general population [30]. An increased incidence of cesareans has been reported [4,54], even if in the previous obstetric literature avoidance of delivery by cesarean was recommended [55,56].

The first study about postnatal survival of children with trisomy 18 was published in 1967: Weber reported a mean survival of 70 days [57]. Most of the ensuing population studies showed a shorter survival, likely because, with prenatal and neonatal diagnosis, it is now possible to diagnose many cases, which would have died prior to detection in the past [3].

Most recent studies report a median survival of 3-14.5 days, a percentage of survival at 24 hours of 60%-75%, at 1 week of 40%-60%, at 1 month of 22%-44%, at 6 months of 9%-18%, and after 1 year of 5%-10% [3,4,6,12,13,15,49,54,58-62]. To summarize, approximately 50% of babies with trisomy 18 live longer than 1 week, and 5-10% of children survive beyond the first year. Because these figures document that 1 in 10 to 1 in 20 babies live to their first birthday, the commonly used term, “lethal abnormality”, is inaccurate, misleading, and inappropriate [12].

The major causes of death are sudden death due to central apnea, cardiac failure due to cardiac malforxmations and respiratory insufficiency due to hypoventilation, aspiration, upper airway obstruction or, likely, the combination of these and other factors [4,12,13,15,49,54,58,59,63-65]. A recent study reported a >100 times higher risk of mortality in neonatal period and in the first years of life for children with trisomy 18 compared to infants born without birth defects [8].

Upper airway obstruction is likely more common than previously realized and should be investigated when full care is opted by the family and medical team. The factors underlying the potential of survival are not known; the presence of heart defects does not seem to affect long-term survival [6]. However a recent trend toward consideration of performing cardiac surgery may alter that premise as surgery may play a role in preventing pulmonary hypertension, a point not investigated in determining the notion that heart defects do not affect survival [6]. A longer survival for females compared to males has been reported, as in the prenatal period [4,6].

Because of the elevated risk of mortality in the first month of life and the presence of significant developmental disability in the surviving children, historically there has been a consensus among care providers that trisomy 18 be considered a condition for which non intervention in the newborn was indicated [65,66]. Nevertheless, the most recent American Academy of Pediatrics neonatal resuscitation guidelines omit trisomy 18 from the list of examples of conditions for which resuscitation is not indicated [67]. A recent survey of the opinion of American neonatologists on newborn care of trisomy 18 infants reported that 44% would intervene mostly because of parental wishes to support the baby [68].

A recent Japanese study documented the survival rate in a group of trisomy 18 newborn to which intensive care were offered: the median survival time (152.5 days) and survival rate at 12 months [25%] were higher compared to those reported in the previous studies, but the survival over 2 years (4%) was similar to the 5-10% usually reported as 1-year survival rate [54]. To our knowledge this is the only study that addresses the question of infant survival if full intervention (short of cardiac surgery) is offered.

In this study the authors also investigated the pathophysiology to death in patients who had intensive treatment; they distinguish between underlying factors associated with death and final modes of death. The common underlying factors associated with death were congenital heart defects and heart failure, and pulmonary hypertension. On the other hand, the final modes of death were sudden cardiac or cardiopulmonary arrest and events related to progressive pulmonary hypertension [54]. From these observations, it becomes clear that apnea and withdrawal of treatment could be considered the major cause of death when a patient with trisomy 18 was managed with purely comfort care. When a patient with trisomy 18 has intensive treatment, the common causes of death are altered, and survival does increase.

The senior author had pointed out in an Editorial [69] in 2006 that there existed a dire need to have a dialogue regarding the ethical issues surrounding the management and care of infants and children with trisomy 18. Such a dialogue seems to be occurring in recent years: the publication of the McGraw and Perlman paper [68] mentioned above and the Ethics Rounds, a Special Article in *Pediatrics* in 2011 [70], both discuss the key themes and controversies that needed current discussion. The former paper indicated that the majority of neonatologists polled in the study would not resuscitate a newborn in the delivery room who had trisomy 18 and a heart defect. The authors stated a concern about a trend away from the “best interest of the child” standard and towards parental opinion. In the more recent Special Article two neonatologists and a parent discuss their views on the management of a baby with trisomy 18 and a heart defect surrounding the decision to have cardiac surgery [70]. While the doctors and the parent disagreed on many points, one of the doctors and the Editor state that “deference to the parents” is generally the best course (unless the child is “suffering” from the ongoing treatment) in situations of unclear outcome. These papers and the published responses to them in *Pediatrics* suggest that a dialogue is in fact now occurring. Another recently published paper by Wilfond and Carey [71], a case-based discussion of the issues and themes involved in the management of trisomy 18 (and related conditions), also illustrates this point of an emerging dialogue. The reader is referred to these papers for further discussion of the relevant issues.

One of the key themes at the center of the controversy is the question of so-called “quality of life” of children and their families when a child has trisomy 18. We will discuss this issue in the Unresolved Questions section below as little data exist in the scientific literature on this topic.

### Growth and feeding

Prenatal growth retardation is one of the most frequent prenatal finding in trisomy 18 [30,35-39]; the mean birth weight is 1700-1800 g at a mean gestational age of 37 weeks [4,54]. Weight and height continue to be below the third centile in the postnatal period; growth charts specific for the condition has been published [49] and are available on the SOFT web pages for the both the US and UK support groups (see Table 1). Head circumference also tends to be below the third centile [12].

Most of the children have feeding difficulties that often require tube feeding in the neonatal period or placement of gastrostomy in the older children (at average age of 8 months) [49]. Both sucking and swallowing problems can be present. Usually the skill of oral feeding if achieved is achieved in infancy, and not later [12,49]. If it is unclear if an infant can or cannot protect her airway, a swallow study can be performed to determine the safety of oral feedings.

Gastroesophageal reflux is a significant medical problem because of both its high prevalence and its potential consequences, like irritability, recurrent pneumonia and aspiration [12]. Aspiration due to gastroesophageal reflux or during feeding is included among the causes of early death [4,12,13,49,54,58,59,61-63].

Gastrointestinal malformations, such as esophageal atresia with tracheo-esophageal fistula, occur with increased frequency but are not a common feature in trisomy 18; pyloric stenosis has been reported and should be considered in the older infant with vomiting [12]. Occasionally the newborn with trisomy 18 can have orofacial clefts that may contribute to feeding problems [12].

### Cardiovascular

Larger series of infants with the syndrome show that 80%-100% of patients with trisomy 18 have congenital structural heart defects; the most common cardiac anomalies are ventricular and atrial septal defects, patent ductus arteriosus and polyvalvular disease [12,72-74].

The majority of the malformations are unlikely to produce neonatal death; this is one of the reasons why the cardiac defect is usually regarded as not causing the early infant mortality. A more complex malformation (double-outlet right ventricle, endocardial cushion defect, or left-sided obstructive lesion) is present in about 10% of cases [12], and then the cardiac defect could play a role in early mortality.

The role of cardiac malformations in causing early death is controversial. Some studies reported that the presence of heart defect does not negatively affect the survival [6] and that the cardiac problems are not implicated in the deaths in most of patients [4]. Based on these data, cardiac surgery in the neonatal period is considered not likely to improve the survival of trisomy 18 children. However, in other studies heart failure and early development of pulmonary hypertension induced by heart defects were found to play a significant role in early death [69,74-76].

Traditionally, heart defects in trisomy 18 patients have been managed conservatively. Recent studies, however, showed that most patients (82-91%) with trisomy 18 can survive palliative and corrective heart surgeries, suggesting that heart surgery can be considered even in patients with trisomy 18 [76-79] (see “Health supervision and management of medical problems” for more details).

### Respiratory

Respiratory problems are one of the most common causes of death in trisomy 18 [4,12,49,54,58,59,61,62]. Pure respiratory problems, such as upper airway obstruction (in some case due to a laryngomalacia or tracheobronchomalacia) and central apnea, can act together with other problems of different origin, like early–onset pulmonary hypertension, feeding difficulties, recurrent aspirations and gastroesophageal reflux, leading to a severe respiratory symptoms [3,4]. Obstructive sleep apnea may be a more common finding in older infants [12] than realized.

### Ophthalmologic

Many ocular findings have been reported in patients with trisomy 18, although major ocular defects are present in a small group of children (less than 10%) [80]. Occasionally, children with trisomy 18 can show anomalies such as a cataract or corneal opacities [81,82]. Short palpebral fissures, visual acuity abnormalities, and photophobia are common findings and underscore the need for ophthalmology assessment in older infants [12]. Photophobia is very common in children with trisomy 18 and requires sunglasses when going outside the home; it likely represents one reason why older infants experience unexplained irritability.

### Ears and hearing

Structural ear anomalies, such as meatal atresia and microtia, are occasionally present. The features of external ear are characteristic: the ear is small with a small lobule, the helix is unfolded, simple and sometimes attached to the scalp (cryptotia) [12]. The ear canal is usually small making audiology screening sometimes challenging. A wide spectrum of middle and internal ear abnormalities has been described. Moderate to severe sensorineural hearing loss can also be present [12].

### Musculoskeletal

Major malformations of limb occur in 5-10% of patients, including radial aplasia and other preaxial limb defects. About 50% of babies show positional foot deformities, both talipes equinovarus and calcaneovalgus. In addition, contractures of other joints can be present explaining why trisomy 18 is sometimes the basis for a neonate labeled artrogryposis. Overriding fingers (second and fifth on third and fourth respectively-see Figure 1) represent one of the important diagnostic clues, often detected sonographically in the prenatal period. Scoliosis is common in older children; usually it is not related to vertebral structural abnormalities and may progress between 5 and 10 years of age [12].

### Genitourinary

Horseshoe kidney is common finding in trisomy 18 (about two-thirds of patients). An increased frequency of urinary tract infections has been observed, perhaps due to structural defects [31]. Otherwise, renal failure is uncommon [12].

### Neoplasia

Trisomy 18 patients have an increased risk to develop some neoplasia, including Wilms tumor and hepatoblastoma [83]. At least 8 cases of Wilms tumor in trisomy 18 children have been reported in the medical literature [83-89]. Nephroblastomatosis, the presence of multiple embryonic rests of tissue within the kidney that may give rise to Wilms tumor, has been detected at autopsy in infants with trisomy 18 who did not die from a Wilms tumor [88-90].

Despite this biological origin, the average age of tumor development is 5 years, ranging from 12 months to 13 years, later than it occurs in general population, suggesting a different biological basis for the tumor in trisomy 18 children [12]. The prognosis is variable.

A child with trisomy 18 has an estimated risk to develop Wilms tumor of about 1% [86]. Because of this high risk, periodic screening with abdominal ultrasound is recommended [48] (see “Health supervision and management of medical problems” for more details).

Seven cases of association between trisomy 18 and hepatoblastoma have been reported [91-97]. The age of diagnosis ranged from 4 months to 3 years. The prognosis was variable: surgical treatment was performed in three patients, two of them were alive without evidence of recurrence at 3 and 4 years of age [93-95], the other died from progression of the tumor [94]. Among the untreated patients, two died of cardiac failure (in one of these hepatoblastoma was an incidental finding at the autopsy) [92-96] and two from progression of the tumor [93,96].

### Neurologic

Several structural abnormalities of the central nervous system have been reported in trisomy 18; the most common are cerebellar hypoplasia, agenesis of corpus callosum, microgyria, hydrocephalus and myelomeningocele, present in about 5% of infants [12,75]. Functional neurologic features include hypotonia in infancy, hypertonia in older children, central apnea and seizures, occurring in 25-50% of children but usually easy to control with pharmacological therapy [12]. Central apnea is one of the principal causes of early death [3,4]. A recent paper described an infant with trisomy 18 and apneic episodes representing complex partial seizures successfully treated with zonisamide [98].

### Developmental and behavior

**Figure 2:**
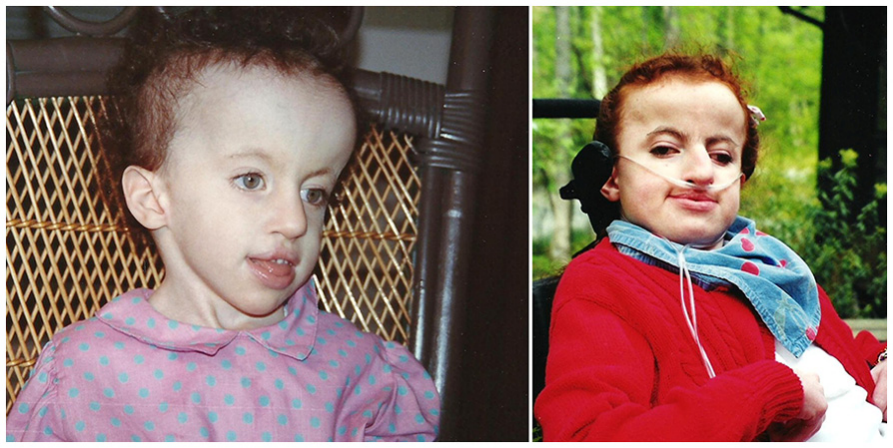
**A young lady with full trisomy 18 in early childhood and in adolescence;she lived to 19 years of age and achieved multiple milestones,including sittingand walking in a walker.**

**Figure 3:**
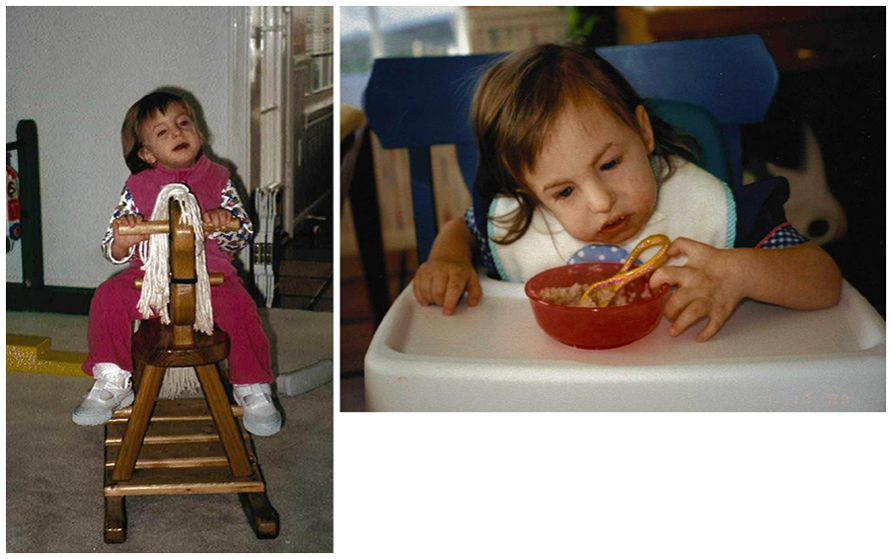
**This girl,now 16 years of age and very healthy,had a ventricular septal defect repair as an infant;she is shown here at various ages enjoying a favorite pastime and feeding herself.** She is walking with assistance but can climb stairs on her own.

In older children with trisomy 18 significant developmental delay is always present ranging from a marked to profound degree of psychomotor and intellectual disability. There is not a regression, but a stable status with slow gaining of some skills. In the most cases expressive language and independently walk are not achieved, but some older children can use a walker [99]. There is also one report of a 4-year-old child with full trisomy 18 who could walk independently [100]. While developmental age in older children is 6-8 months overall, most have some skills of older children, including sleeping independently, self-feeding, imitating, using a sign board, following simple command, and understanding cause and effect [99]. All children acquire abilities such as recognizing their family and smiling appropriately [99]. (See Figures 2 and 3). Recognizing the significant delays, Baty et al., state in their article describing developmental skills in older children with trisomy 18 (and 13) “ Older children could use a walker, understand words and phrases, use a few words or signs, crawl, follow simple commands, recognize and interact with others and play independently” [99]. Thus children with trisomy 18, while showing marked developmental and cognitive disability have many more abilities than usually perceived in the stereotype and prior portrayals of the condition (Figures 2 and 3).

As mentioned above (see “Etiology and Pathogenesis”) among patients with mosaic trisomy 18 the phenotype is extremely variable, and there is no correlation between the percentage of trisomy 18 cells in either blood cells or skin fibroblasts and the severity of intellectual disabilities [24].

## Health supervision and management

After the discharge from the hospital, follow-up visits for health supervision should be regular and often in the first weeks and months of life; referral to the appropriate pediatric subspecialists can occur. In the long-survival children, the frequency of health supervision visits may decrease as they advance, depending on the specific needs of each child.

Generally, children with trisomy 18 should receive the same routine care, e.g., anticipatory guidance and immunizations that all children receive. In regards to administering immunizations, the weight and overall status of an infant, in particular the presence of a seizure disorder, should be taken into consideration. Decisions surrounding the treatment of specific problems should be decided upon with the parents and medical team according to the degree of the involvement and what is in the best interest of the child [49].

Table 3 summarizes the schedule of clinical and laboratory/referrals at the time of birth or diagnosis and during the follow up periods. These are modeled after other recent guidelines for the routine care of children with rare diseases.

**Table 3:** Guidelines for routine evaluation in children with trisomy 18 at time of diagnosis and during follow up

**Area of clinical evaluation**	**Time**	**Assessment**
Growth and feeding	every visit	Use published growth curves, investigate need for enteral nutrition
Psychomotor and cognitive developmental progress	every visit	developmental delay and referral to early intervention program and PT/OT
Neurologic exam	every visit	muscular tone abnormalities, seizures, referral to neurology if needed
Cardiology and echocardiogram	at birth/diagnosis – follow up as needed	congenital heart defect, pulmonary hypertension
Abdominal ultrasound	at birth/diagnosis - follow up as needed	renal malformation
	every 6 months until adolescence	Wilms tumor and hepatoblastoma
Ophthalmology	at birth/diagnosis	eye malformations
	older children	photophobia and refractive defects, prescribe sunglasses as needed
Audiology	at birth/diagnosis - follow up as needed	sensorineural hearing loss
Orthopedic exam	every visit in children older than 2 years	scoliosis
Gastroenterology	if needed	gastroesophageal reflux, need of enteral nutrition
Pulmonology	if needed	recurrent pulmonary infections, central and obstructive apnea
Sleep study	if needed	central and obstructive apnea

Adapted from Carey [12].

### Growth and feeding

Growth parameters (weight, length and head circumference) should be checked during each evaluation, more frequently in the first weeks and months of life, and plotted on the specific growth charts [49].

Assessment of the sucking or swallowing problems with a radiographic swallow study can be useful if needed to consider the ability of the child to protect the airway; use of feeding tube in neonatal period or placement of gastrostomy can be considered to assure appropriate and safe feeding. Referral to a feeding or dysphagia team is an option.

Gastroesophageal reflux should be considered as a potential factor in feeding problems. If needed, standard medical therapy may be started. If medical treatment is not successful, surgery can be considered [12].

### Cardiovascular

At the time of diagnosis or in the newborn period cardiac evaluation including echocardiogram should be performed. Traditionally, heart defects in trisomy 18 patients have been managed conservatively. Since 1990s few reports of cardiac surgery in this population has been published [49,58], but recently four studies on larger series of patients appeared in the medical literature [76-79]. These investigations showed that most patients (82-91%) with trisomy 18 can survive palliative and corrective heart surgeries and can be discharged from the hospital [76,77]. In one study from Japan the median postoperative survival reported was 179 days, and the median survival for this group of patients was 324 days [76]. In the same study, the most frequent cause of death was infections; otherwise heart failure was the cause of death in only one patient, suggesting that cardiac surgery is effective in preventing congenital heart defect-related death [76]. Therefore, the authors concluded that intensive care, including optional cardiac surgery, in selected patients with trisomy 18 is ethically acceptable [79].

In a recent investigation Yamagishi et al., [78] suggests that surgery should be considered in trisomy 18 infants because it may improve life expectancy, facilitate discharge from the hospital, and improve quality of life of both patient and family. The author qualifies the recommendation in stating that the risk of surgery in patients with trisomy 18 is higher than in patients without trisomy 18 or in patients with trisomy 21, and acknowledges that it is still unknown whether the cardiac surgery improves the long-term prognosis of trisomy 18 children.

Recently Maeda et al. reported the results of a nationwide questionnaire-based study made by the Japanese Society of Pediatric Cardiology and Cardiovascular Surgery [79]. They collected and evaluated clinical data from 134 patients with trisomy 18: 94% of patients had congenital heart defects, the most frequent one was ventricular septal defect (59%) and 52% of patients developed pulmonary hypertension. Twenty-five percent of patients with congenital heart defects underwent cardiac surgery, and 56% of these patients have survived beyond postoperative period. In most patients palliative surgery was performed, but 19% of children underwent intracardiac repair for ventricular septal defect. Operated patients survived longer than those who did not have surgery.

The severity of cardiac defect and the indications for pharmacological or surgical treatment differ among patients with trisomy 18. Therefore, individual evaluation considering the overall health state of the infant is needed to determine optimal treatment [78].

These above stated approaches and views that lead to the option of cardiac surgery are controversial as reflected in the paper cited above by Janvier et al. [70] and in the more recently comprehensive review of the topic by Merritt et al. [101]. In this latter article the authors summarize much of the previous literature on the ethical and legal aspects of care and recommend a palliative care model in the care of infants with trisomy 18 (and trisomy 13). We will discuss this theme more below in the section on Unresolved Questions.

### Respiratory

Evaluation by a pulmonologist can be performed if respiratory problems become important, especially in the infant where it is difficult to sort out the various factors that might be playing a role, i.e., upper airway obstruction, pulmonary hypertension and central apnea. Evaluations do not differ from those in other children with similar symptoms. Sleep study can be useful to detect the severity of sleep apnea problems. Decisions about home monitoring and oxygen therapy should be made with parents on an individual basis [12].

In recent years there appears to be an increase in therapeutic procedures including tracheostomy placement in children with trisomy 18 [102] (see Figure 1). Also of note the web page of the Support Organization of Trisomy 18, 13, and Related Disorders, maintains and updates a registry of surgical procedures (including heart and tracheostomies) documented in children with trisomy 18 ( http://www.trisomy.org). As in all decision-making in the care of infants with trisomy 18, parents and physicians make these choices when the intervention is in the best interest of the child.

Administration of palivizumab for the prevention of RSV lower respiratory tract disease should be considered in infants with trisomy 18 even those without congenital heart defects.

### Ophthalmologic

Ophthalmologic evaluation is recommended to detect common structural abnormalities and, in older children, visual acuity defects [12]. When needed, treatment of eye defects is the same as in other children. In older infants with photophobia sunglasses are usually helpful.

### Ears and hearing

Audiological evaluation is recommended in all infants; if sensorineural hearing loss is detected, the use of hearing aids can be offered and attempted [12].

### Musculoskeletal

In children older than 2 years, clinical evaluation of the spine should be performed at each health supervision visit, followed by spine X-ray and specialist evaluation if scoliosis is clinically suspected. Sometimes, in older children, surgery for severe scoliosis should be considered because of consequent restrictive lung disease.

The decision about treatment of clubfoot in infants (with cast or surgery) is complex, because only a small percentage of children with trisomy 18 can walk assisted or independently.

### Genitourinary

Abdominal ultrasound screening is recommended in children with trisomy 18.

If renal abnormalities are detected, follow up for urinary infection and renal failure by periodic blood and urine analysis should be performed. The treatment of urinary infections does not differ from that in any other child.

### Neoplasia

The high incidence of intra-abdominal tumors, particularly Wilms tumor and hepatoblastoma, in trisomy 18 children justifies the recommendation of abdominal sonographic screening in these patients. There is no established timing for the screening, but it may be started after 6 months of life with a screening every 6 months and continued into adolescence because one of the cases of Wilms tumor reported developed in a 13-year-old female [12,87].

### Neurologic

Neurological evaluation is recommended in all trisomy 18 patients. Usually they need physical therapy for tone muscle abnormalities. Management of epilepsy is similar to that in other children; seizures are generally well controlled by standard pharmacological therapy.

### Developmental and behavior

At each health supervision visits assessment of developmental progression through standard developmental evaluation is mandatory, and early referral to intervention programs and physical therapy is recommended.

### Overall care and ongoing support

The key ingredient in carrying out effective health supervision in the care of infants and children with trisomy 18 is a committed primary care practitioner. As pointed out by Carey [48] a clinician who is willing to oversee the care and provide ongoing support to the family should not be hesitant to take on the challenge of shepherding the management of a child with this disorder (despite its relative rareness) and providing the Medical Home for the children. Additionally referral to a palliative care team can aid in the needed ongoing support and be a good resource for the family and clinician.

## Unresolved questions

As mentioned above the most clearly unresolved issue is the controversy surrounding the option of aggressive respiratory or surgical treatment of infants with trisomy 18. In this concluding section we will try to provide some perspective on this highly complex topic.

Because of the high neonatal and infant mortality and because of the issue usually described as the quality of life in children with the syndrome, many practitioners in the US and Europe have argued for a noninterventionist approach with accompanying comfort care (sometimes called custodial) and currently with the guidance of a palliative care team [101]. This view was articulated by Bos et al and Paris et al. [65,66] and discussed in detail most recently by Janvier et al. [70] and Merritt et al. [101] as mentioned above. The conventional view at least among US neonatologists is reflected in the survey study by McGraw and and Perlman where 55% of the physicians polled stated that they would not resuscitate a newborn in the delivery room known to have trisomy 18 and a ventricular septal defect [68]. The Ethics Rounds paper by Janvier et al. [70] comprehensively summarizes all of the themes that emerge in any discussion of care in a baby with trisomy 18. These themes include the following: the best interest of the child standard, parent autonomy, allocation of resources, quality of life of children with trisomy 18, and the potential pain and suffering experienced if treatment occurs for the child. In the recent paper by Merritt et al. [101], the authors provide an even more referenced review of the important themes of the topic. These authors present a list of questions to consider in the setting of a prenatal and postnatal diagnosis of trisomy 18 (and 13). They close their paper with a poignant assertion, “We assert that transforming hope for cure to hope for the child and the family to be relieved from suffering, and to experience love and care in their infant’s lifetime, should be the primary goal.” The authors could not agree more. However, Merritt et al. consistently use the descriptor “lethal” throughout the paper. They, like the authors of many of their cited papers, perceive trisomy 18 as “lethal” when in fact at least 1 in 20 infants survive the first year of life even with modern day approaches, which tend to be comfort care and non-intervention [71].

In an Invited Comment Kosho [103] reflected on the varied views in Japan on the care of infants with trisomy 18. This author summarized a series of guidelines for parents and providers in determining choices around the medical care of serious newborn conditions of which trisomy 18 represents the prototype. These guidelines along with the tables published in the Merritt et al. paper [101] go a long way in initiating needed dialogue and guidelines on this theme.

What is missing in both the Janvier et al. Ethics Rounds [70] and in the Merritt et al. treatise [101] are two themes: 1. clarification of 5-8% infant survival, (which is clearly documented in the figures from multiple population studies cited in the this paper), and 2. the complete picture of quality of life, which is known only from perusal of the parents support group websites [see Table 2, SOFT US and UK], the papers by Baty et al., [49,101], and the recent article by Janvier et al. where the authors report parents’ positive experiences in rearing a child with trisomy 18 (and 13) [104].

Let us reiterate these themes: 1. As indicated 5-8% of infants with trisomy 18 without special care live to their first birthday; thus as pointed out by Wilfond and the senior author (JCC), “lethal” is a misplaced and misleading description [70]; 2. Parents and families of children with trisomy 18 cope well, appreciate a unique quality of life in their children, value their children deeply, and want to be a part of the decisions made around care. In a study of a web-based survey Janvier et al. documented this experience in over 300 families coping with the challenges of parenting children with trisomy 18 internationally [105]. Fenton [105] also related his experience as a palliative care specialist and expanded the traditionally narrowed view of quality of life in children and their parents with trisomy 18 (and 13). Bruns [106] articulated these themes in a recent article that reports on parent-reported data.

There is no simple solution to this dilemma and controversy. Certainly more qualitative and quantitative data on the experience of families are needed. Their voice - while reflecting one aspect of the whole portrait - is crucial and vital. Secondly, international consensus on guidelines for care that includes all of the specialties involved in the care of fetuses, newborns, and older children with trisomy 18 is required. (The authors are currently organizing such a consensus group). Thirdly, as suggested by Wilfond and Carey [73], avoidance of the use of the term “lethal.” Continuation of the now ongoing dialogue on this topic by pediatricians, geneticists, bioethicists, families, and the appropriate care specialists is mandatory and welcomed. In a recent opinion piece one of us (JCC) reviewed this emerging dialogue surrounding treatment issues in trisomy 18 (and 13); the reader is referred to that paper for additional discussion of the themes and controversies [107].

## Competing interest

The authors declare that they have no competing commercial interests that would influence the writing of this paper.

## Authors' contributions

The authors contributed equally to the preparation of this paper. Both authors read and approved the final manuscript.

## References

[B1] Edwards JH Harnden DG Cameron AH Crosse VM Wolff OH A new trisomic syndrome Lancet 1960 1 787 789 13819419 10.1016/s0140-6736(60)90675-9

[B2] Smith DW Patau K Therman E Inhorn SL A new autosomal trisomy syndrome: multiple congenital anomalies caused by an extra chromosome J Pediatr 1960 57 338 345 10.1016/S0022-3476(60)80241-7 13831938

[B3] Root S Carey JC Survival in trisomy 18 Am J Med Genet 1994 49 170 174 10.1002/ajmg.1320490203 8116664

[B4] Embleton ND Wyllie JP Wright MJ Burn J Hunter S Natural history of trisomy 18 Arch Dis Child 1996 75 38 41 10.1136/fn.75.1.f38 PMC1061148 8795354

[B5] Forrester MB Merz RD Trisomies 13 and 18: prenatal diagnosis and epidemiologic studies in Hawaii, 1986-1997 Genet Test 1999 3 335 340 10.1089/gte.1999.3.335 10627940

[B6] Rasmussen SA Wong L Yang Q May K Friedman JM Population-based analyses of mortality in trisomy 13 and trisomy 18 Pediatrics 2003 111 777 784 10.1542/peds.111.4.777 12671111

[B7] Crider KS Olney RS Cragan JD Trisomies 13 and 18: population prevalences, characteristics, and prenatal diagnosis, metropolitan Atlanta, 1994-2003 Am J Med Genet 2008 146A 820 826 10.1002/ajmg.a.32200 18348276

[B8] Parker SE Mai CT Canfield MA Rickard R Wang Y Meyer RE Anderson P Mason CA Collins JS Kirby RS Correa A National birth defects prevention network. Updated national birth prevalence estimates for selected birth defects in the United States, 2004-2006 Birth Defects Res A Clin Mol Teratol 2010 88 1008 1016 10.1002/bdra.20735 20878909

[B9] Irving C Richmond S Wren C Longster C Embleton ND Changes in fetal prevalence and outcome for trisomies 13 and 18: a population-based study over 23 years J Matern Fetal Neonatal Med 2011 24 137 141 10.3109/14767051003758879 20384468

[B10] Morris JK Savva GM The risk of fetal loss following a prenatal diagnosis of trisomy 13 or trisomy 18 Am J Med Genet 2008 146A 827 832 10.1002/ajmg.a.32220 18361449

[B11] Won RH Currier RJ Lorey F Towner DR The timing of demise in fetuses with trisomy 21 and trisomy 18 Prenat Diagn 2005 25 608 611 10.1002/pd.1243 16032775

[B12] Carey JC Cassidy SB, Allanson JE Trisomy 18 and trisomy 13 syndromes Management of genetic syndromes 2010 3 John Wiley & Sons, New York 807 823

[B13] Carter PE Pearn JH Bell J Martin N Anderson NG Survival in trisomy 18 Clin Genet 1985 27 59 61 3978839 10.1111/j.1399-0004.1985.tb00184.x

[B14] Young ID Cook JP Mehta L Changing demography of trisomy 18 Arch Dis Child 1986 61 1035 1936 10.1136/adc.61.10.1035 3777987 PMC1777964

[B15] Goldstein H Nielsen KG Rates and survival of individuals with trisomy 18 and 13 Clin Genet 1988 34 366 372 3233784 10.1111/j.1399-0004.1988.tb02894.x

[B16] Kupke KG Mueller U Parental origin of the extra chromosome in trisomy 18 Am J Hum Genet 1989 45 599 605 2577470 PMC1683505

[B17] Fisher JM Harvey JF Morton NE Jacobs PA Trisomy 18: studies of the parent and cell division of origin and the effect of aberrant recombination on nondisjunction Am J Hum Genet 1995 56 669 675 7887421 PMC1801162

[B18] Eggermann T Nothem MM Eiben B Hofmann JD Hinkel K Fimmers R Schwanitz G Trisomy of human chromosome 18: molecular studies on parental origin and cell stage of nondisjunction Hum Genet 1996 97 218 223 10.1007/BF02265269 8566957

[B19] Bugge M Collins A Petersen MB Fisher J Brandt C Hertz JM Tranebjaerg L DeLozier-Blanchet C Nicolaides P Brondum-Nielsen K Morton N Mikkelsen M Non-disjunction of chromosome 18 Hum Mol Genet 1998 7 661 669 10.1093/hmg/7.4.661 9499419

[B20] Hassold TJ Burrage LC Chan ER Judis LM Schwartz S James SJ Jacobs PA Thomas NS Maternal folate polymorphisms and the etiology of human nondisjunction Am J Hum Genet 2001 69 434 439 10.1086/321971 11443546 PMC1235315

[B21] Savva GM Walker K Morris JK The maternal age-specific live birth prevalence of trisomies 13 and 18 compared to trisomy 21 (down syndrome) Prenat Diagn 2010 30 57 64 19911411 10.1002/pd.2403

[B22] De Souza E Morris JK EUROCAT Working Group Case–control analysis of paternal age and trisomic anomalies Arch Dis Child 2010 95 893 897 10.1136/adc.2009.176438 20584846

[B23] Bettio D Levi Setti P Bianchi P Grazioli V Trisomy 18 mosaicism in a woman with normal intelligence Am J Med Genet 2003 120A 303 304 10.1002/ajmg.a.20213 12833422

[B24] Tucker ME Garringer HJ Weaver DD Phenotypic spectrum of mosaic trisomy 18: two new patients, review of the literature and counseling issues Am J Med Genet 2007 143A 505 517 10.1002/ajmg.a.31535 17266111

[B25] Beratis NG Hsu LY Kutinsky E Hirschhorn K Stability of trisomic [47, 18þ] cells in long-term mosaic skin fibroblast culture Can J Genet Cytol 1972 15 869 870 4653581 10.1139/g72-106

[B26] Gersdorf E Utermann B Utermann G Trisomy 18 mosaicism in an adult woman with normal intelligence and history of miscarriage Hum Genet 1990 84 298 299 2303251 10.1007/BF00200581

[B27] Ukita M Hasegawa M Nakahori T Trisomy 18 mosaicism in a woman with normal intelligence, pigmentary dysplasia, and an 18 trisomic daughter Am J Med Genet 1997 68 240 241 10.1002/(SICI)1096-8628(19970120)68:2<240::AID-AJMG24>3.0.CO;2-T 9028467

[B28] Wilson GN Karyotype/phenotype correlation: prospects and problems illustrated by trisomy 18 The Phenotypic Mapping of Down Syndrome and Other Aneuploid Conditions 1993 Wiley-Liss, New York 157 173 8115400

[B29] Boghosian-Sell L Mewar R Harrison W Shapiro RM Zackai EH Carey JC David L Keppen L Hudgins L Overhauser J Molecular mapping of the Edwards syndrome phenotype to two noncontiguous regions on chromosome 18 Am J Hum Genet 1994 55 476 483 8079991 PMC1918415

[B30] Yamanaka M Setoyama T Igarashi Y Kurosawa K Itani Y Hashimoto S Saitoh K Takei M Hirabuki T Pregnancy outcome of fetuses with trisomy 18 identified by prenatal sonography and chromosomal analysis in a perinatal center Am J Med Genet 2006 140 1177 1182 16652360 10.1002/ajmg.a.31241

[B31] Staples AJ Robertson EF Ranieri E Ryall RG Haan EA A maternal serum screen for trisomy 18: an extension of maternal serum screening for down syndrome Am J Hum Genet 1991 49 1025 1033 1833973 PMC1683263

[B32] Perni SC Predanic M Kalish RB Chervenak FA Chasen ST Clinical use of first-trimester aneuploidy screening in a United States population can replicate data from clinical trials Am J Obstet Gynecol 2006 194 127 130 10.1016/j.ajog.2005.06.068 16389021

[B33] Breathnach FM Malone FD Lambert-Messerlian G Cuckle HS Porter TF Nyberg DA Comstock CH Saade GR Berkowitz RL Klugman S Dugoff L Craigo SD Timor-Tritsch IE Carr SR Wolfe HM Tripp T Bianchi DW D’Alton ME First, second trimester evaluation of risk (FASTER) research consortium: first- and second-trimester screening: detection of aneuploidies other than down syndrome Obstet Gynecol 2007 110 651 657 10.1097/01.AOG.0000278570.76392.a6 17766613

[B34] Sherod C Sebire NJ Soares W Snijders RJ Nicolaides KH Prenatal diagnosis of trisomy 18 at the 10-14-week ultrasound scan Ultrasound Obstet Gynecol 1997 10 387 390 10.1046/j.1469-0705.1997.10060387.x 9476321

[B35] Sepulveda W Wong AE Dezerega V First-trimester sonographic findings in trisomy 18: a review of 53 cases Prenat Diagn 2010 30 256 259 20112232 10.1002/pd.2462

[B36] Geipel A Willruth A Vieten J Gembruch U Berg C Nuchal fold thickness, nasal bone absence or hypoplasia, ductus venosus reversed flow and tricuspid valve regurgitation in screening for trisomies 21, 18 and 13 in the early second trimester Ultrasound Obstet Gynecol 2010 35 535 539 10.1002/uog.7597 20183867

[B37] Hill LM The sonographic detection of trisomies 13, 18, and 21 Clin Obstet Gynecol 1996 39 831 850 10.1097/00003081-199612000-00011 8934034

[B38] Viora E Zamboni C Mortara G Stillavato S Bastonero S Errante G Sciarrone A Campogrande M Trisomy 18: fetal ultrasound findings at different gestational ages Am J Med Genet 2007 143A 553 557 10.1002/ajmg.a.31615 17318852

[B39] Cho RC Chu P Smith-Bindman R Second trimester prenatal ultrasound for the detection of pregnancies at increased risk of Trisomy 18 based on serum screening Prenat Diagn 2009 29 129 139 10.1002/pd.2166 19142904

[B40] Inagaki M Ando Y Mito T Ieshima A Ohtani K Takashima S Takeshita K Comparison of brain imaging and neuropathology in cases of trisomy 18 and 13 Neuroradiology 1987 29 474 477 10.1007/BF00341747 3317111

[B41] Twinning P Zuccollo J Clewes J Swallow J Fetal choroid plexus cysts: a prospective study and review of the literature Br J Radiol 1991 64 98 102 10.1259/0007-1285-64-758-98 1825933

[B42] Gross SJ Shulman LP Tolley EA Emerson DS Felker RE Simpson JL Elias S Isolated fetal choroid plexus cysts and trisomy 18: a review and meta-analysis Am J Obstet Gynecol 1995 172 83 87 10.1016/0002-9378(95)90088-8 7847564

[B43] Shields LE Uhrich SB Easterling TR Cyr DR Mack LA Isolated fetal choroid plexus cysts and karyotype analysis: is it necessary? J Ultrasound Med 1996 15 389 394 8731447 10.7863/jum.1996.15.5.389

[B44] Reinsch RC Choroid plexus cysts-association with trisomy: prospective review of 16,059 patients Am J Obstet Gynecol 1997 176 1381 1383 10.1016/S0002-9378(97)70363-6 9215202

[B45] Demasio K Canterino J Ananth C Fernandez C Smulian J Vintzileos A Isolated choroid plexus cysts in low-risk women less than 35 years old Am J Obstet Gynecol 2002 187 1246 1249 10.1067/mob.2002.127463 12439513

[B46] Beke A Barakonyi E Belics Z Joo JG Csaba A Papp C Toth-Pal E Papp Z Risk of chromosome abnormalities in the presence of bilateral or unilateral choroids plexus cysts Fetal Diagn Ther 2008 23 185 191 10.1159/000116739 18417976

[B47] Niedrist D Riegel M Achermann J Rousson V Schinzel A Trisomy 18: changes in sex ratio during intrauterine life Am J Med Genet 2006 140A 2365 2367 10.1002/ajmg.a.31474 17022073

[B48] Carey JC Health supervision and anticipatory guidance for children with genetic disorders (including specific recommendations for trisomy 21, trisomy 18, and neurofibromatosis I) Pediatr Clin N Am 1992 39 40 43 10.1016/s0031-3955(16)38261-x 1531255

[B49] Baty BJ Blackburn BL Carey JC Natural history of trisomy 18 and trisomy 13. I. Growth, physical assessment, medical histories, survival, and recurrence risk Am J Med Genet 1994 49 175 188 10.1002/ajmg.1320490204 8116665

[B50] Uehara S Yaegashi N Maeda T Hoshi N Fujimoto S Fujimori K Yanagida K Yamanaka M Hirahara F Yajima A Risk of recurrence of fetal chromosomal aberrations: analysis of trisomy 21, trisomy 18, trisomy 13, and 45, X in 1,076 Japanese mothers J Obstet Gynaecol Res 1999 25 373 379 10.1111/j.1447-0756.1999.tb01180.x 10680333

[B51] De Souza E Halliday J Chan A Bower C Morris JK Recurrence risks for trisomies 13, 18, and 21 Am J Med Genet 2009 149A 2716 2722 10.1002/ajmg.a.33099 19921649

[B52] Jones KL Smith’s Recognizable Patterns of Malformation 2008 6 Phila, WB Saunders/Elsevier

[B53] Simpson JL German J Developmental anomaly resembling the trisomy 18 syndrome Ann Genet 1969 12 107 110 5308380

[B54] Kosho T Nakamura T Kawame H Baba A Tamura M Fukushima Y Neonatal management of trisomy 18: clinical details of 24 patients receiving intensive treatment Am J Med Genet 2006 140A 937 944 10.1002/ajmg.a.31175 16528744

[B55] Schneider AS Mennuti MT Zackai EH High cesarean section rate in trisomy 18 births: a potential indication for late prenatal diagnosis Am J Obstet Gynecol 1981 140 367 370 7246651 10.1016/0002-9378(81)90028-4

[B56] Rochelson BL Trunca C Monheit AG Baker DA The use of a rapid in situ technique for third-trimester diagnosis of trisomy 18 Am J Obstet Gynecol 1986 155 835 836 3766638 10.1016/s0002-9378(86)80032-1

[B57] Weber WW Survival and the sex ratio in trisomy 17-18 Am J Hum Genet 1967 19 369 377 6026929 PMC1706214

[B58] Van Dyke DC Allen M Clinical management considerations in long-term survivors with trisomy 18 Pediatrics 1990 85 753 759 2330236

[B59] Niedrist D Riegel M Achermann J Schinzel A Survival with trisomy 18–data from Switzerland Am J Med Genet 2006 140A 952 959 10.1002/ajmg.a.31172 16528741

[B60] Imataka G Nitta A Suzumura H Watanabe H Yamanouchi H Arisaka O Survival of trisomy 18 cases in Japan Genet Couns 2007 18 303 308 18019371

[B61] Vendola C Canfield M Daiger SP Gambello M Hashmi SS King T Noblin SJ Waller DK Hecht JT Survival of Texas infants born with trisomies 21, 18, and 13 Am J Med Genet 2010 152A 360 366 10.1002/ajmg.a.33156 20082470

[B62] Chen CP Su YN Hsu CY Ling PY Tsai FJ Chern SR Wu PC Chen HE Wang W Second-trimester molecular prenatal diagnosis of sporadic apert syndrome following sonographic findings of mild ventriculomegaly and clenched hands mimicking trisomy 18 Taiwan J Obstet Gynecol 2010 49 129 132 10.1016/S1028-4559(10)60028-9 20466312

[B63] Matthews AL Chromosomal abnormalities: Trisomy 18, trisomy 13, deletions, and microdeletions J Perinat Neonatal Nurs 1999 13 59 75 10818854 10.1097/00005237-199909000-00006

[B64] Yang Q Chen H Correa A Devine O Mathews TJ Honein MA Racial differences in infant mortality attributable to birth defects in the United States, 1989–2002 Birth Defects Res A Clin Mol Teratol 2006 76 706 713 10.1002/bdra.20308 17022030

[B65] Paris JJ Weiss AH Soifer S Ethical issues in the use of life-prolonging interventions for an infant with trisomy 18 J Perinatol 1992 12 366 368 1282542

[B66] Bos AP Broers CJM Hazebroek FWJ Van Hemel JO Tibboel D Swaay EW Molenaar JC Avoidance of emergency surgery in newborn infants with trisomy 18 Lancet 1992 339 913 917 10.1016/0140-6736(92)90940-5 1348308

[B67] American Heart Association 2005 American heart association (AHA) guidelines for cardiopulmonary resuscitation (CPR) and emergency cardiovascular care (ECC) of pediatric and neonatal patients: pediatric advanced life support Pediatrics 2006 117 e1005 e1028 16651281 10.1542/peds.2006-0346

[B68] McGraw MP Perlman JM Attitudes of neonatologists toward delivery room management of confirmed trisomy 18: potential factors influencing a changing dynamic Pediatrics 2008 121 1106 1110 10.1542/peds.2007-1869 18519479

[B69] Carey JC Introductory comments on special section: trisomy 18 Amer J Med Genet 2006 140A 935 936 10.1002/ajmg.a.31251

[B70] Janvier A Okah F Farlow B Lantos JD Ethics rounds: an infant with trisomy 18 and a ventricular septal defect Pediatrics 2011 127 754 759 10.1542/peds.2010-1971 21402635

[B71] Wilfond BS Carey JC Diekema D, Mercurio MR, Adam MB Parental requests for interventions in children with lethal conditions Clinical Ethics in Pediatrics: A Case-Based Textbook 2011 Cambridge University Press Cambridge, Cambridge 174 180

[B72] Van Praagh S Trumen T Firpo A Bano-Rogriga A Freid R McManus B Engle MA Van Praagh R Cardiac malformations in trisomy-18: a study of 41 postmortem cases J Am Coll Cardiol 1989 13 1586 1597 10.1016/0735-1097(89)90353-7 2723271

[B73] Balderston SN Schaffer EN Washington RL Sondheimer HM Congenital polyvalvular disease in trisomy 18: echocardiographic diagnosis Pediatr Cardiol 1990 11 138 142 10.1007/BF02238843 2395741

[B74] Musewe NN Alexander DJ Teshima I Smalhorn JF Freedom RM Echocardiographic evaluation of the spectral cardiac anomalies associated with trisomy 18 and 13 J Am Coll Cardiol 1990 15 673 677 10.1016/0735-1097(90)90644-5 2303637

[B75] Kinoshita M Nakamura Y Nakano R Fukuda S Thirty-one autopsy cases of trisomy 18: clinical features and pathological findings Pediatr Pathol 1989 9 445 457 10.3109/15513818909022365 2798270

[B76] Kaneko Y Kobayashi J Yamamoto Y Yoda H Kanetaka Y Nakajima Y Endo D Tsuchiya K Sato H Kawakami T Intensive cardiac management in patients with trisomy 13 or trisomy 18 Am J Med Genet 2008 146A 1372 1380 10.1002/ajmg.a.32311 18412275

[B77] Graham EM Bradley SM Shirali GS Hills CB Atz A Pediatric Cardiac Care Consortium Effectiveness of cardiac surgery in trisomies 13 and 18 (from the pediatric cardiac care consortium) Am J Cardiol 2004 93 801 803 10.1016/j.amjcard.2003.12.012 15019900

[B78] Yamagishi H Cardiovascular surgery for congenital heart disease associated with trisomy 18 Gen Thorac Cardiovasc Surg 2010 58 217 219 10.1007/s11748-009-0501-6 20449710

[B79] Maeda J Yamagishi H Furutani Y Kamisago M Waragai T Oana S Kajino H Matsuura H Mori K Matsuoka R Nakanishi T The impact of cardiac surgery in patients with trisomy 18 and trisomy 13 in Japan Am J Med Genet A 2011 155A 2641 2646 21990245 10.1002/ajmg.a.34285

[B80] Geiser SC Carey JC Apple DJ Human chromosomal disorders and the eye Goldberg’s Genetic and Metabolic Eye Disease 1986 Little, Brown, Boston

[B81] Calderone JP Chess J Borodic G Albert DM Intraocular pathology of trisomy 18 (Edwards syndrome): report of a case and review of the literature Br J Ophthalmol 1983 67 162 169 10.1136/bjo.67.3.162 6824623 PMC1040001

[B82] Geiser CF Schindler AM Long term survival in a male with 18- trisomy and Wilms’ tumor Pediatrics 1969 44 111 116 4307567

[B83] Karayalcin G Shanske A Honigman R Wilms’ tumor in a 13-year old girl with trisomy 18 Am J Dis Child 1981 135 665 666 6264780 10.1001/archpedi.1981.02130310069024

[B84] Wang-Wuu S Soukup S Bove K Gotwals B Lampkin B Chromosome analysis of 31 Wilms’ tumors Cancer Res 1990 50 2786 2793 2158398

[B85] Faucette KJ Carey JC Trisomy 18 and Wilms’ tumor—is there an association? Clin Res 1991 39 96A

[B86] Carey JC Faucette KJ Schimke RN Increased risk of Wilms tumor in children with trisomy 18: the evidence and recommendations for a surveillance protocol Proc Greenwood Genet Cent 2002 21 74

[B87] Anderson CE Punnett HH Huff V de Chadarevian JP Characterization of a Wilms tumor in a 9-year-old with trisomy 18 Am J Med Genet 2003 121A 52 55 10.1002/ajmg.a.20141 12900902

[B88] Bove KE Koffler H McAdams AJ Nodular renal blastema, definition, and possible significance Cancer 1969 24 323 332 10.1002/1097-0142(196908)24:2<323::AID-CNCR2820240215>3.0.CO;2-K 4307750

[B89] Shanklin DR Sotello-Avilla C In situ tumors in fetuses, newborns, and infants Biol Neonate 1969 14 286 316 10.1159/000240196 4315735

[B90] Olson JM Hamilton A Breslow NE Non-11p constitutional chromosome abnormalities in Wilms tumor patients Med Pediatr Oncol 1995 24 305 309 10.1002/mpo.2950240507 7700182

[B91] Dasouki M Barr MJ Trisomy 18 and hepatic neoplasia Am J Med Genet 1987 27 203 205 10.1002/ajmg.1320270122 3037903

[B92] Mamlok V Nichols M Lockhart L Mamlok R Trisomy 18 and hepatoblastoma Am J Med Genet 1989 33 125 126 10.1002/ajmg.1320330119 2546426

[B93] Tanaka K Uemoto S Asonuma K Katayama T Utsunomiya H Akiyama Y Sasaki MS Ozawa K Hepatoblastoma in a 2-year-old girl with trisomy 18 Eur J Pediatr Surg 1992 2 298 300 10.1055/s-2008-1063464 1329941

[B94] Bove KE Soukup S Ballard ET Rychman F Hepatoblastoma in a child with trisomy 18: cytogenetics, liver anomalies, and literature review Pediatr Pathol Lab Med 1996 16 253 262 10.1080/107710496175732 9025831

[B95] Teraguchi M Nogi S Ikemoto Y Ogino H Kohdera U Multiple hepatoblastomas associated with trisomy 18 in a 3- year-old girl Pediatr Hematol Oncol 1997 14 463 467 10.3109/08880019709028777 9267879

[B96] Maruyama K Ikeda H Koizumi T Hepatoblastoma associated with trisomy 18 syndrome: a case report and a review of the literature Pediatr Int 2001 43 302 305 10.1046/j.1442-200x.2001.01380.x 11380930

[B97] Kitanovski L Ovcak Z Jazbec J Multifocal hepatoblastoma in a 6-month-old girl with trisomy 18: a case report J Med Case Reports 2009 3 8319 10.4076/1752-1947-3-8319 PMC2726543 19830224

[B98] Kumada T Nishii R Higashi T Oda N Fujii T Epileptic apnea in a trisomy 18 infant Pediatr Neurol 2010 42 61 64 10.1016/j.pediatrneurol.2009.08.004 20004866

[B99] Baty BJ Blackburn BL Carey JC Natural history of trisomy 18 and trisomy 13. II. Psychomotor development Am J Med Genet 1994 49 189 194 10.1002/ajmg.1320490205 7509567

[B100] Ray S Ries MD Bowen JR Arthrokatadysis in trisomy 18 Pediatr Orthop 1986 6 100 101 10.1097/01241398-198601000-00020 3941168

[B101] Merritt DA Catlin A Wool C Paverini R Goldstein M Oshiro B Trisomy 18 and trisomy 13: treatment and management decisions NeoReviews 2012 3 E40 E48

[B102] Nelson KE Hexem KR Feudtner C Inpatient hospital care of children with trisomy 3 and trisomy 18 in the United States Pediatrics 2012 129 869 876 10.1542/peds.2011-2139 22492767

[B103] Kosho T Invited comment: care of children wit trisomy 18 in Japan Amer J Med Genet Part A 2008 146A 1369 1371 10.1002/ajmg.a.32355 18470922

[B104] Janvier A Farlow B Wilfond B The experience of families with children with trisomy 3 and 18 in social networks Pediatrics 2012 130 293 298 10.1542/peds.2012-0151 22826570

[B105] Fenton LJ Trisomy 18 and 13: quality of life: treading “softly” Amer J Med Genet A 2011 155 1527 1528 10.1002/ajmg.a.34084 21671397

[B106] Bruns DA Neonatal experiences of newborns with full trisomy 18 Adv Neonatal Care 2010 10 25 31 10.1097/ANC.0b013e3181cbf54e 20150778

[B107] Carey JC Perspectives on the care and management of infants with trisomy 18 and trisomy 13: striving for balance Curr Opinions in Pediatrics 2012 in press 10.1097/MOP.0b013e3283595031 23044555

